# The long noncoding RNA LINC00341 suppresses colorectal carcinoma by preventing cell migration and apoptosis

**DOI:** 10.1002/cbf.3473

**Published:** 2020-02-17

**Authors:** Shuyuan Li, Shuo Chen, Boxue Wang, Lin Zhang, Yinan Su, Xipeng Zhang

**Affiliations:** ^1^ Department of Colorectal Surgery Tianjin Union Medical Center Tianjin China

**Keywords:** apoptosis, colorectal carcinoma, HMGB2, LINC00341, lncRNA, migration

## Abstract

Long noncoding RNAs (lncRNAs) are ubiquitous transcripts that play key roles in regulating gene expression at the levels of transcription, RNA processing, and translation. Aberrant expression and mutations of lncRNAs represent a driving force behind oncogenesis and development of tumours. However, most of the lncRNAs are still being undiscovered, and conclusive experimental evidence for their functional relevance continues to be lacking for most malignancies. We have found that lncRNA long intergenic non–protein‐coding RNA 341 (LINC00341) is aberrantly downregulated by microarray‐based screenings on nonmetastatic and metastatic colorectal carcinoma (CRC) specimens; LINC00341 is a novel long intergenic non–protein‐coding RNA with unknown functions. LINC00341 overexpression restricts tumour growth and promotes its apoptosis. Instead, LINC00341 silencing accelerates CRC cell proliferation and migration. RNA‐pulldown assay identifies LINC00341 physically binds to HMGB2 and stabilizes the localization of HMGB2 in the cytoplasm. Notably, LINC00341 knockdown leads to the shift of HMGB2 into nuclear, in which it triggers epithelial to mesenchymal transition (EMT) programming. Moreover, LINC00341 can also promote apoptosis.

**Significance of the study:**

LncRNAs are ubiquitous transcripts that play key roles in regulating gene expression at the levels of transcription, RNA processing, and translation. Aberrant expression and mutations of lncRNAs represent a driving force behind oncogenesis and development of tumours. However, the function of lncRNA still needs further exploration. Our study has revealed a new noncoding RNA‐mediated regulatory network that highly likely protects colorectal carcinoma by preventing migration and apoptosis. The results will help further explore the molecular details about the progression of colorectal carcinoma and stimulate efforts to develop effective therapies.

## 1 INTRODUCTION

Colorectal carcinoma (CRC) is currently the third most common malignancy and the second leading cause of cancer‐related death worldwide.^1^ Despite the advancement of surgery, radiation, and chemotherapy, the 5‐year survival rate of CRC patients has not significantly improved during the past decades.^2^, ^3^ For malignant tumours, early detection, diagnosis, and treatment can greatly improve patient survival.^3^ Conventional diagnostic methods are difficult to identify tumours less than 1 cm, and the sensitivity and specificity of traditional tumour markers are not sufficient. The occurrence of CRC involves many factors and complex steps, and the corresponding biomarkers are needed to be explored.^4^

Long noncoding RNAs (lncRNAs) are a group of non–protein‐coding transcripts that are longer than 200 nucleotides.^5^, ^6^, ^7^ Despite of fast turnover rates and low copy numbers, lncRNAs have been widely accepted as truly functional biomolecules.^8^ Recent studies have shown that long‐chain noncoding RNA (lncRNAs) plays a major role in human cancer.^9^ Their discovery and research have changed our understanding of cancer cell biology to a certain extent. At present, some lncRNAs are thought to activate tumour suppressor genes or oncogenes in common cancers and participate in the occurrence and development of cancers,^10^ and their molecular mechanisms are also elucidated. Nevertheless, there are still many unknown or controversial functional mechanisms of lncRNAs at the forefront of cancer research.^11^ It is imperative to further study its identification, function, and mechanism, which may be brilliant in the basic and clinical research on cancer. LINC00341 is a novel long intergenic non–protein‐coding RNA with unknown functions. In this report, we demonstrate that lncRNA long intergenic non–protein‐coding RNA 341 (LINC00341) is aberrantly downregulated in CRC. Low expression of LINC00341 promoted patient's poor survival, as well as cancer metastasis. Epithelial to mesenchymal transition (EMT) is widely regarded as a key step for CRC to acquire an invasive or metastatic phenotype. It has been reported that TGFβ triggers EMT process by dampening E‐cadherin and upregulating vimentin in multiple CRC cell lines.^12^ Recent studies have also shown that lncRNAs activated by TGFβ is upregulated in serum of CRC patients, and EMT is induced by reducing the expression of epithelial markers E‐cadherin, ZO‐1, and increasing the expression of interstitial markers ZEB1 and N‐cadherin (n‐cad).^13^, ^14^ In our study, we concluded that LINC00341 can bind to HMGB2 to inhibit tumour migration. In this report, we also demonstrate that partially restoring the level of LINC00341 could attenuate the survival of cultured CRC cells by promoting apoptosis. Nowadays, no reports are available regarding the biological function of LINC00341. Our report first suggested that LINC00341 may represent a potential biomarker in colorectal cancer.

## 2 RESULTS

### 2.1 LINC00341 expression was decreased in colorectal cancer

**Figure 1:**
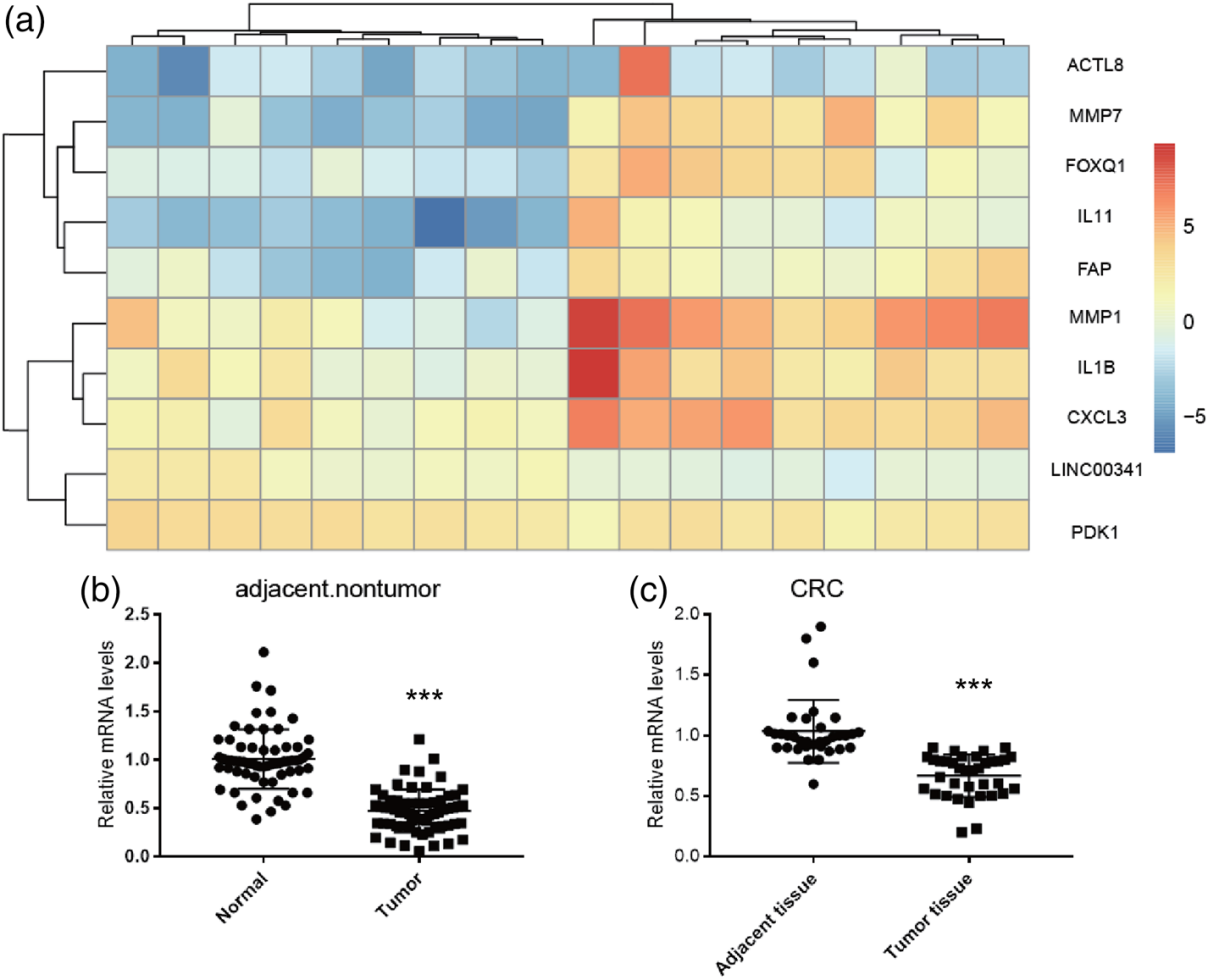
Differential expression of LINC00341 in colorectal cancer. A, Heat map and hierarchical clustering (log2 fold change and *q* value <0.05) are presented to show the variation in lncRNAs between paired colorectal cancer and adjacent nontumor colorectal tissues. Red indicates high expression, and green indicates low relative expression. B, LINC00341 expression in normal samples and CRC samples was measured by qRT‐PCR. C, LINC00341 expression in 36 paired with colorectal carcinoma tissues was determined using real‐time PCR assays, compared with the adjacent tissues. LINC00341 expression level was normalized to GAPDH. Results are shown as means ± SD by two‐tailed Student's *t* test.  *P<0.01. **P<0.001. ***P<0.0001

To identify transcripts that potentially drive CRC metastasis, lncRNA and messenger RNA (mRNA) expression profiles were determined by microarray analysis. The microarray data were deposited in NCBI Gene Expression Omnibus and are accessible through GEO series accession number GSE104836. A total of 1019 lncRNAs (512 upregulated and 507 downregulated) and 3221 mRNAs (1606 upregulated and 1615 downregulated) were differentially expressed in tumour colorectal tissues (fold change > 2 and *P* < .05). Hierarchical clustering showed systematic variations in the expression of lncRNAs and protein‐coding RNAs between nontumor colorectal tissues and tumour colorectal tissues (Figure 1A). Next, total RNAs were isolated from the colorectal tissues, and RNA levels were determined by qRT‐PCR. We found that the levels of lncRNA LINC00341 in the colorectal tissues were much lower in tumour colorectal tissues (Figure 1B). To validate this result, we also performed quantitative real‐time PCR. Total RNAs were isolated from the adjacent colorectal tissues and colorectal tissues. We can also find that LINC00341 is downregulated (Figure 1C).

### 2.2 Decreased LINC00341 expression correlated with tumour progression and poor prognosis of CRC patients

**Figure 2:**
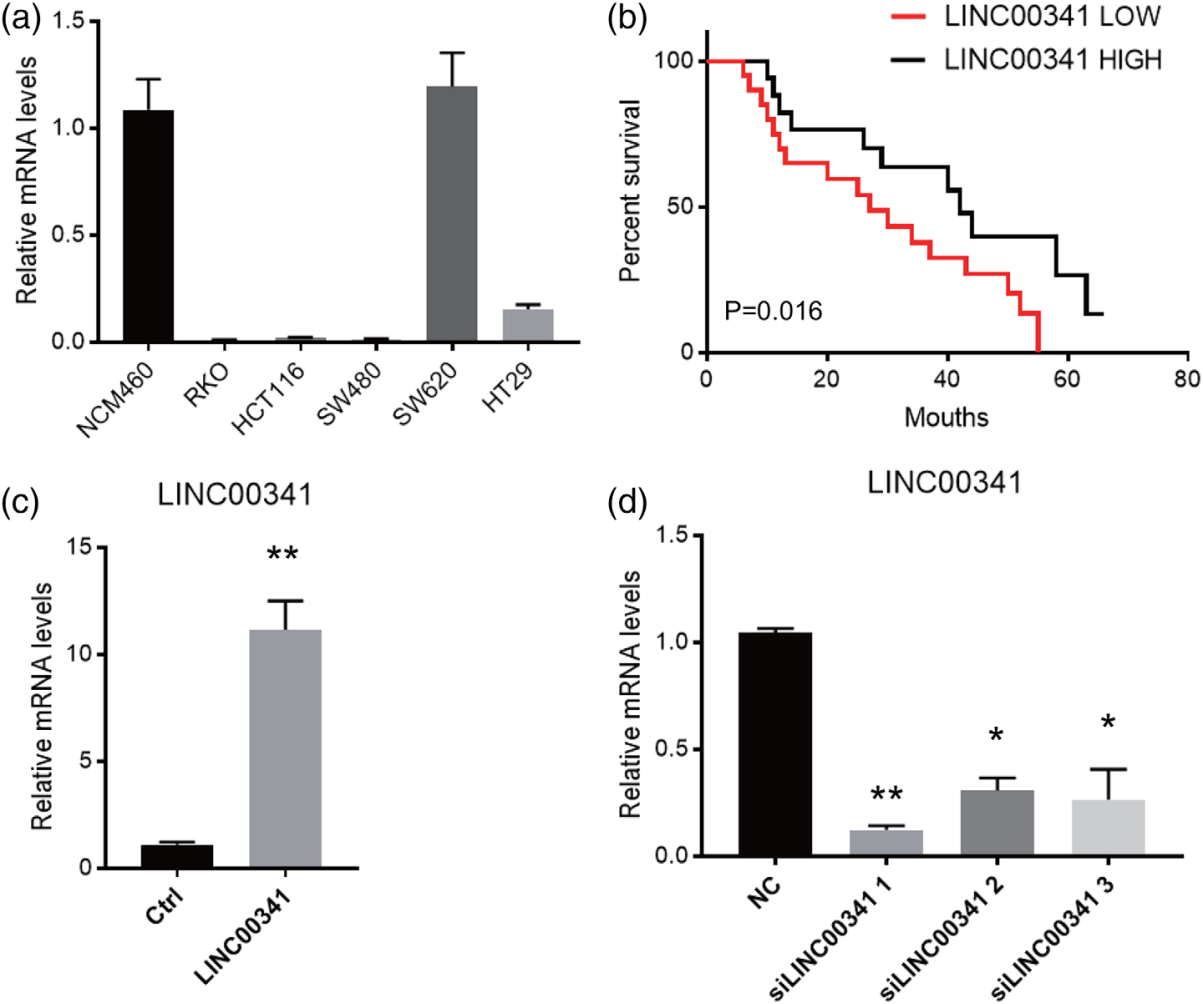
Gain and loss function in vitro of LINC00341. A, Expression levels of LINC00341 in five CRC cell lines. NCM460 was invoked as a normal control. Data are shown as means ± SD. Data are representative of three independent experiments. B, Kaplan‐Meier overall survival curves for 20 patients with BC classified according to relative LINC00341 expression level. C, Overexpression of LINC00341 in cultured HCT116. The LINC00341 bar represents the level of the lncRNA in the cells transfected with a LINC00341‐overexpressing lentivirus, while NC represents the level of the lncRNA in the cells transfected with a negative‐control lentivirus. D, SW620 cells were transfected with three siRNA for LINC00341 to knock down LINC00341 expression. LINC00341 expression level was normalized to GAPDH. Results are shown as means ± SD by two‐tailed Student's *t* test. *P < .001. **P < .01. ***P < .001

Based on the decreased LINC00341 expression in colorectal cancer, we next evaluate the relationship between LINC00341 and CRC progression, and we analysed the correlation between high LINC00341 expression and clinicopathological features of CRC; the data are summarized in (Table 1). No significant association was found between LINC00341 expression and age (*P* = .534), gender (*P* = .362), and location (*P* = .364). However, LINC00341 expression significantly correlated with TNM stage (*P* = .012), differentiation (*P* = .021), AJCC Stage I/II (*P* = .041), AJCC Stage III/IV (*P* = .009), and lymph node metastasis (*P* = .018). Next, we evaluated the prognostic effect of LINC00341 on overall survival by comparing the overall survival of CRC patients with high or low LINC00341 levels. A total of 34 paired cases of CRC patients were divided into two groups: a high LINC00341 expression group (above the median LINC00341 expression, n = 17) and a low LINC00341 expression group (below the median LINC00341 expression, n = 17). Among the participants, patients with high LINC00341 expression were associated with a significantly higher survival rate than those with a low expression, according to Kaplan‐Meier curve assessment (*P* = .016, log‐rank test; Figure 2B). The above findings suggested that a decreased LINC00341 expression was significantly correlated with progression, metastasis, and poor outcome in CRC patients.

**Table 1:** Correlation between LINC00341 and the clinicopathological parameters of colorectal carcinoma

	Total Number (n = 63)		Expression of LINC00341		
Features			Low Expression	High Expression	*P* values
Age, y	<60	12	3	9	*P* = .534
	≥60	51	12	39	
Gender					
	Male	30	8	22	*P* = .362
	Female	31	11	20	
TNM stage					
	Tri, T1, and T2	5	4	1	*P* = .012
	T3 and T4	58	17	41	
Location					
	C, A, T	32	12	20	*P* = .364
	D, S, R	29	11	18	
Differentiation					
	Well	5	3	2	*P* = .021
	Moderate	43	15	28	
	Poor	15	1	14	
AJCC Stage					
	I	6	4	2	*P* = .041
	II	28	9	19	
	III	18	5	13	*P* = .009
	IV	13	4	9	
Lymph node metastasis					
	No	45	16	29	*P* = .018
	Yes	18	5	13	
Tumour size					
	<3 cm	3	2	1	*P* = .654
	≥3 cm	60	17	43	

### 2.3 Downregulated and upregulated LINC00341 in CRC cells in vitro

The expression of LINC00341 was analysed in five CRC cell lines and a normal human intestinal mucosal cell line NCM460. As shown in Figure 2A, lower expression of LINC00341 was found in all CRC cell lines,^15^ except SW620 cells, in comparison with NCM460. To evaluate the effects of LINC00341 on cell biological behaviours, gain and loss functions in vitro were performed. HCT116 was infected with the lentivirus containing the LINC00341 overexpression vector (Figure 2C). On the contrary, SW620 cells were transfected with siRNA for LINC00341 to knock down LINC00341 expression. Among the three siRNA, we select the most efficient one for subsequent experiments (Figure 2D).

### 2.4 LINC00341 inhibits aggressive phenotypes and reverses EMT process of CRC cells in vitro

**Figure 3:**
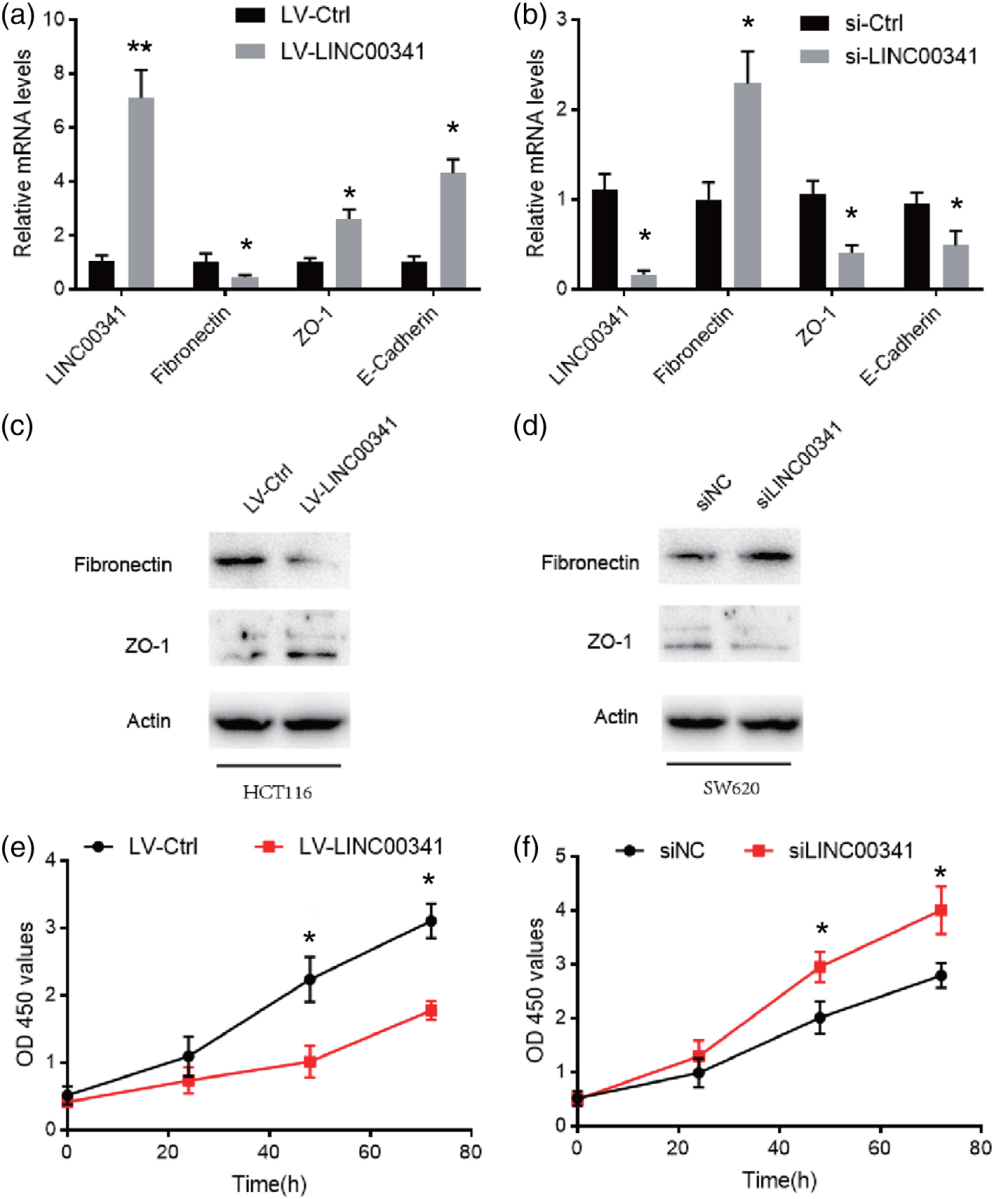
LINC00341 overexpression inhibits tumorigenic and metastasis of CRC in vitro. A, Real‐time PCR analysis of epithelial to mesenchymal transition (EMT) markers expression of LINC00341 overexpression in HCT116. B, Real‐time PCR analysis of EMT markers expression of LINC00341 knockdown in SW620. C, Western blot analysis was carried out to detect EMT markers of LINC00341 overexpression in HCT116. D, Western blot analysis was performed to detect EMT markers of LINC00341 knockdown in SW620. E, The CCK8 assay used to evaluate the proliferation of the HCT116 cells after transfection with the LINC00341 lentivirus or control. F The CCK8 assay used to evaluate the proliferation of the SW620 cells after transfection with the LINC00341 siRNA or control. Error bars represent mean ± SD based on three independent experiments. Results are shown as means ± SD by two‐tailed Student's *t* test. *P < .001. **P < .01. ***P < .001

To evaluate the effects of LINC00341 on cell biological behaviours, gain and loss functions in vitro were performed. HCT116 were infected with the lentivirus containing the LINC00341 overexpression vector. On the contrary, SW620 cells were transfected with siRNA for LINC00341 to knock down LINC00341 expression. The initiation of EMT is the driving force for tumour invasion and metastasis. LINC00341 overexpression upregulated mRNA expression of epithelial markers E‐cadherin and ZO‐1. In contrast, LINC00341 silencing downregulated their mRNA expression as expected. Besides, overexpression of LINC00341 causes downregulation of mesenchymal marker fibronectin. As expected, LINC00341 silencing upregulated fibronectin mRNA expression (Figure 3A,B). The protein expression of epithelial markers ZO‐1 and mesenchymal marker fibronectin was further confirmed in HCT116 and SW620 cells (Figure 3C,D). CCK‐8 proliferation assay showed that cell proliferation was decreased and HCT116 was infected with the lentivirus containing the LINC00341 overexpression vector. Which compared with transfected cells (Figure 3E). Conversely, SW620 cells were transfected with siRNA for LINC00341 to knock down LINC00341 expression significantly promoted the cell proliferation of the SW620 (Figure 3F). As showed above, LINC00341 strikingly reduced the potential of cell migration, invasion, and motility, respectively.

### 2.5 LINC00341 promotes cell apoptosis process of CRC cells in vitro

**Figure 4:**
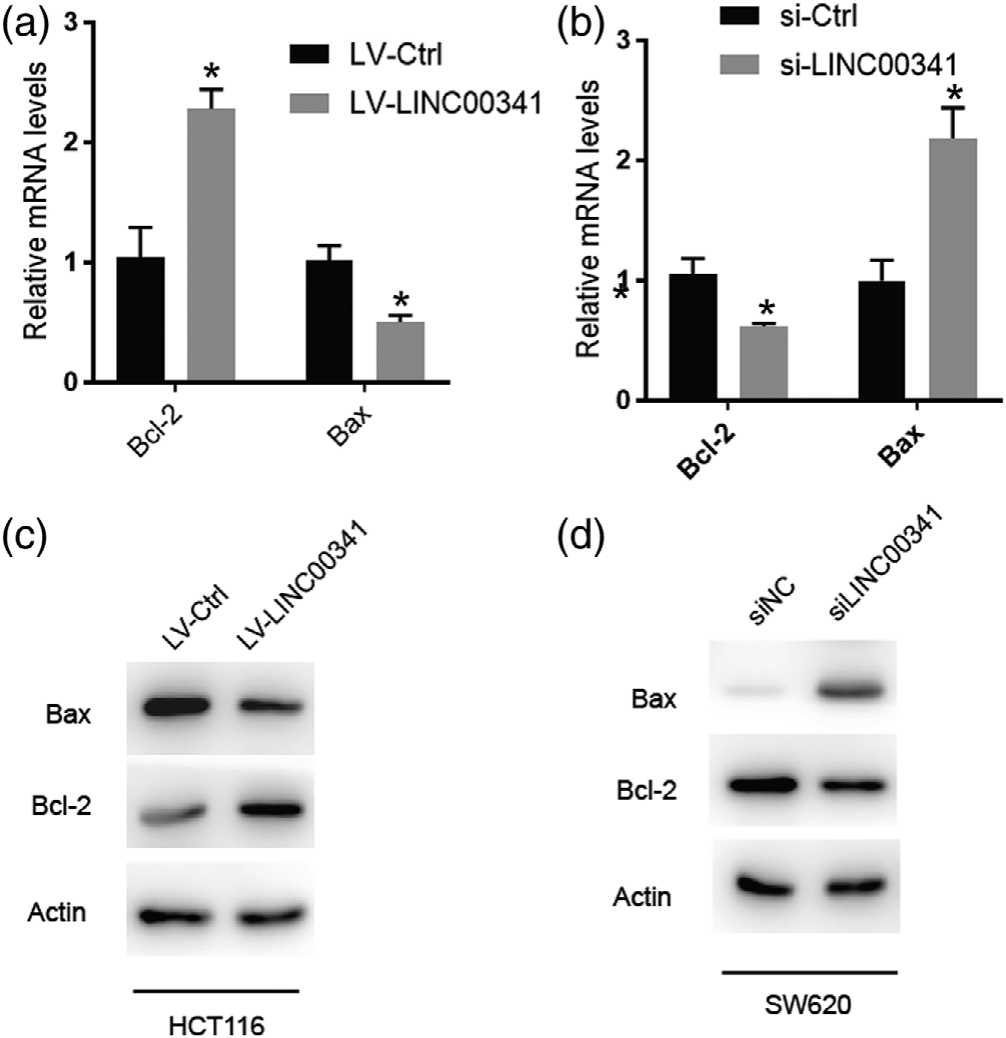
LINC00341 overexpression promotes apoptosis of colorectal carcinoma (CRC) in vitro. A, Real‐time PCR analysis of apoptosis markers expression of LINC00341 overexpression in HCT116. B, Real‐time PCR analysis of apoptosis markers expression of LINC00341 knockdown in SW620. C, Western blot analysis was performed to detect apoptosis markers of LINC00341 overexpression in HCT116. D, Western blot analysis was performed to detect apoptosis markers of LINC00341 knockdown in SW620. Error bars represent mean ± SD based on three independent experiments. Results are shown as means ± SD by two‐tailed Student's *t* test. *P < .001. **P < .01. ***P < .001

We found that the levels of lncRNA LINC00341 in the CRC tissue were much lower than those in the normal tissues (Figure 1B). This suggested that deregulation of LINC00341 is involved in the development of the disease. To investigate the cellular function of LINC00341, we tried to express this lncRNA in cultured CRC cells and address the impact. The HCT116 cells overexpressing LINC00341 resulting in increased expression of Bcl‐2 and decreased expression of Bax, indicating an increase in higher apoptosis level (Figure 4A). Conversely, knockdown the expression of LINC00341 resulting in decreased expression of Bcl‐2 and increased expression of Bax. indicating an increase in lower apoptosis level (Figure 4B). We probed two proteins involved in apoptosis and found that overexpressing LINC00341 downregulated the proapoptotic protein Bax but upregulated the antiapoptotic protein B‐cell lymphoma 2 (Bcl‐2). The opposite result can also be seen in CRC cells which knock down the expression of LINC00341 (Figure 4C,D). The results suggested that LINC00341 is probably required to inhibit the progression of CRC.

### 2.6 LINC00341 interacts with HMGB2 protein and regulates its nucleocytoplasmic shuttling

**Figure 5:**
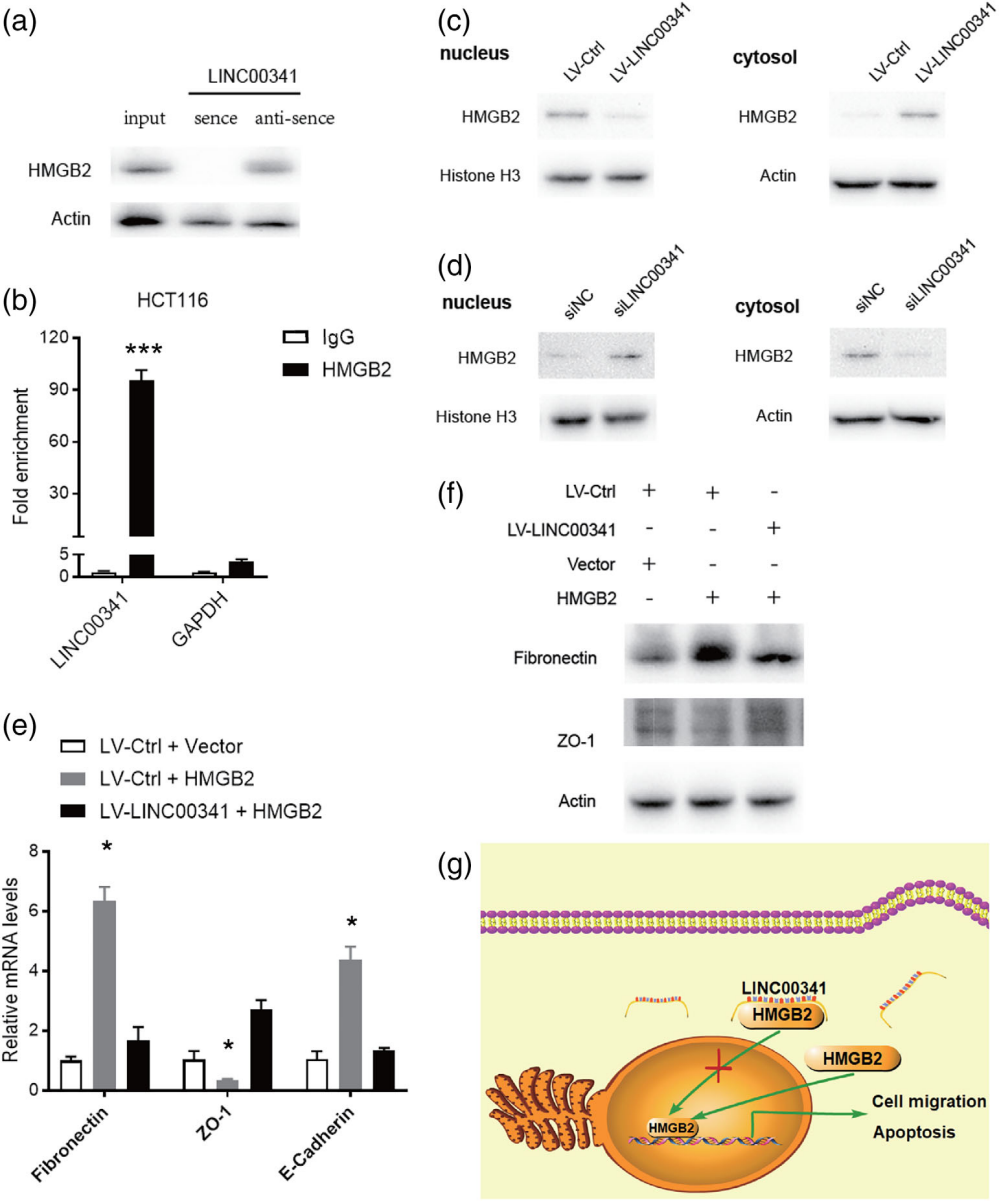
LINC00341 interacts with HMGB2 and suppress it into the nucleus. A, RNA pulldowns were performed with SW620 cells using full‐length LINC00341 transcript sense and antisense. HMGB2 was confirmed by immunoblotting. B, Interaction of LINC00341 with HMGB2 was verified by an RNA immunoprecipitation (RIP) assay. C,D, The expression of HMGB2 in nucleus and cytoplasm of LINC00341 overexpressing HCT116 cells and LINC00341‐knockdown SW620 cells were demonstrated by Western blot. The immunosignal was quantified using the quantity one software. Nuclear segregation is assayed by Histone H3. Cytoplasmic segregation is assayed by Actin. E, Real‐time PCR analysis of epithelial to mesenchymal transition (EMT) markers expression infected with LINC00341 lentivirus or control lentivirus and transfection of HMGB2 vector and empty vector. F, Western blot analysis was performed to detect EMT markers. G, A hypothetical model illustrating that LINC00341 suppresses colorectal cancer progression through nucleocytoplasmic shuttling of HMGB2. Error bars represent mean ± SD based on three independent experiments. Results are shown as means ± SD by two‐tailed Student's t test. *P < .001. **P < .01. ***P < .001

HMGB2 is overexpressed in CRC and contributes CRC progression. HMGB2 is a key EMT‐associated transcription factors. To seek proteins that are interacted with LINC00341, RNA‐pulldown assay was conducted in SW620 cells. RNA‐associated proteins were identified by SDS‐PAGE and subjected to mass spectrometry. Among all proteins identified by mass spectrometry, HMGB2 was successfully validated by Western blot with RNA‐pulldown analysis. After pulldown operation with the sense sequence of LINC00341, significant enrichment of HMGB2 was detected (Figure 5A). But no enrichment was observed with the antisense LINC00341 as blotted by HMGB2 antibody. We further use the HMGB2 antibody as a bait to carry out RNA immunoprecipitation (RIP) with cell extracts from the SW620 tumour cell lines. We observed that the LINC00341 enrichment, with GAPDH mRNA, remains unchanged, using the HMGB2 antibody vs a nonspecific antibody (IgG control) (Figure 5B). HMGB2 is a nuclear protein that binds to DNA and plays a role in chromatin remodelling. LINC00341 overexpression significantly decreased its level in the nucleus and increased the level of HMGB2 in the cytoplasm. In contrast, LINC00341 silencing led to the enrichment of HMGB2 in the nuclear (Figure 5C,D). To further prove the interaction of LINC00341 with HMGB2. We overexpressed HMGB2 at the cellular level, and we found HMGB2 can upregulate mRNA expression E‐cadherin and fibronectin, however, the mRNA expression of ZO‐1 was decreased. Meanwhile, we further overexpress LINC00341. As we expected, the increase E‐cadherin and fibronectin range of HMGB2 is weakened due to the overexpression of LINC00341. Under previous results, LINC00341 reversed the decrease of ZO‐1 mRNA level caused by overexpression of HMGB2 (Figure 5E). The same results were verified on Western blot (Figure 5F). So we came to this conclusion. LINC00341 can interact with HMGB2 reverses EMT process of CRC cells in vitro.

## 3 DISCUSSION

LncRNA imbalance has been found in many human cancers, including CRC, which is the third most common cancer in the world with poor prognosis. Besides, there is strong evidence that lncRNAs can affect tumour suppressor genes or oncogenes.^16^ LncRNAs play important roles in gene regulation and affect many aspects of cellular homeostasis,^9^, ^17^ and they are closely related to tumour occurrence and development.^4^, ^18^ Using the microarray scanning and bioinformatics analyses, we identified a potential tumour metastasis suppressor in CRC, designated as LINC00341. In previous studies, however, it has been demonstrated that some transcripts annotated as lncRNAs are encoding for small proteins.^19^ Linc00341 full‐length cDNA was subcloned into two eukaryotic expression vectors with N‐terminal or C‐terminal markers. No protein expression was observed (results were not presented). In patients with colorectal cancer, the expression of LINC00341 is not only correlated with superior survival outcomes but also negatively correlated with advanced clinical stage. Then, a series of in vitro experiments provided consolidated data of Linc00341 inhibiting the growth and metastasis of colorectal cancer, suggesting that LINC00341 is a potent inhibitor of colorectal cancer metastasis.

High‐mobility group box (HMGB) proteins 1, 2, and 3 function as universal sentinels for nucleic acids. HMGBs bind to all immunogenic nucleic acids examined with a correlation between affinity and immunogenic potential.^15^ Abnormal expression and localization of HMGB protein were found in various tumours. Increased expression of HMGB2 in patients with hepatocellular carcinoma was significantly associated with shortened overall survival time.^7^, ^20^ Compared with adjacent normal breast tissues, HMGB2 is highly expressed in the nucleus of breast cancer cells.^21^ In our study, LINC00341 was combined with HMGB2 to form an inhibitory complex, stabilizing the location of HMGB2 cell formation. Therefore, LINC00341 usually promotes the retention of HMGB2 in the cytoplasm. Our study shows that the reduced expression of Linc00341 in SW260 CRC cells will allow HMGB2 transfer to the nucleus. Suggesting that following up, we found that LNC can promote cell apoptosis, but the specific molecular mechanism is still unclear. LINC00341 may be an important regulator of HMGB2, regulating the nuclear shuttle of HMGB2. It has been reported that HMGB2 binds to DNA without sequence specificity and promotes transcription by expanding the accessibility of chromatin to transcription factors.^22^ HMGB2 interacts with OCT4 to maintain multifunctional gene expression in mouse embryonic stem cells.^23^ Oct 4 that was activated by TGFβ1 can trigger EMT and switch cells to a more stem cell‐like phenotype in bladder cancer.^24^

In recent years, many studies have shown that lncRNAs were associated with the occurrence and development of various tumours. Among them, lncRNAs have been increasingly reported being involved in colorectal cancer.^25^ However, the expression and biological function of lncRNA LINC00341 in colorectal cancer is not illuminated. In our study, we first found that the LINC00341 level in colorectal cancer was significantly lower than that in normal adjacent tissues. Overexpression of LINC00341 can suppress the proliferation and invasion/migration and promote apoptosis in CRC cells. In summary, LINC00341 is an effective tumour suppressor, which is related to a better prognosis of CRC patients. By stabilizing the cytoplasmic localization of HMGB2, it can play an antimetastasis role. Furthermore, we found that LINC00341 can promote cell apoptosis, but the molecular biological mechanism of LINC00341 has not been well studied, and the underlying molecular mechanism needs further study.

## 4 MATERIALS AND METHODS

### 4.1 Patients and microarray analysis

Surgical removal of CRC from patients were selected from the Tianjin Union Medical Center (Tianjin, China). The tissues were snap‐frozen in liquid nitrogen and then stored at −80°C. This project was approved by the Ethics Committee of Tianjin Union Medical Center. The gene expression data have been assigned an accession ID as GSE113296. The standard selection criteria to identify differentially expressed genes are as follows: (a) log2 fold change ≥1.5 or <0.5 and (b) *P* < .05.

### 4.2 Cell culture

CRC cell lines HCT116, SW480, SW620, RKO, and HT29 and normal human colon epithelial cell line NCM460 were obtained from the American Type Culture Collection (ATCC). RPMI‐1640 medium (Invitrogen, Carlsbad, CA) was used to culture the indicated cells, added 10% fetal bovine serum (FBS, Gibco, Carlsbad, CA), incubated in a 37°C humidified atmosphere of 5% CO_2_. LINC00341 overexpression lentiviral vector and lentiviral vectors were all purchased from ViGene (Shangdong, China). Transfection of LINC00341 siRNA. The siRNA sequences used in this study were LINC00341‐1 5′UUACGUAUAAACGAAAGUAAA3′, LINC00341‐2 5′UUAGAACCUUUUACUCCUAGA3′, LINC00341‐3 5′UUGUAAUUUUGUGUCCGAGCA3′. Lipofectamine 2000 reagent was purchased from Invitrogen (Carlsbad, California, USA). CRC cells at exponential growth phase were plated into 6‐well plates for 24 hours at a density of 0.5 × 10^5^ cells/mL and transfected according to the manufacturer's protocol.

### 4.3 RNA isolation and quantitative RT‐PCR

Total RNA was extracted from mouse liver or primary hepatocytes using a Trizol‐based method (Roche Molecular Biochemicals, Indianapolis, IN). Approximately 2 μg of total RNA was reverse‐transcribed into a first‐strand cDNA pool using SuperScript reverse transcriptase and random primers (Abcam). Q‐PCR was performed using the SYBR Green I Q‐PCR kit (Promega) with a Bio‐Rad CFX system. All gene expression data were normalized to GAPDH expression levels. Primers for Q‐PCR are listed below.

human LINC00341 5′GCAGGACTCAGCATCTCCCA3′(forward), 5′CTCGGCTGGACAAGGTGGTT3′ (reverse).
human ZO‐1 5′TCATCTCCAGTCCCTTACCT3′ (forward), 5′GCTCCTCCAGTCTGACATTAG3′ (reverse).
human Fibronectin 5′TCACCCTCACCAACCTCACT3′ (forward), 5′CATCCCAGCTGATCAGTAGGC3′(reverse).
human E‐cadherin 5′TGGCTTCCCTCTTTCATCTC3′ (forward), 5′ACTTTAGGCACTATTCTAAGT3′ (reverse).
human GAPDH 5′CATCAAGAAGGTGGTGAAGCA3′ (forward), 5′TCAAAGGTGGAGGAGTGGGT3′ (reverse).

### 4.4 Cell Counting Kit‐8 assay

Cells viability was quantitated with a Cell Counting Kit‐8 (CCK‐8) solution (Dojindo, Kumamoto, Japan). Briefly, cells were seeded in a 96‐well plate at 2 × 10^3^ cells per well. After incubation for 24, 48, 72, and 96 hours, 10‐μL CCK‐8 solution was added to each well, and the cells were further incubated for 4 hours. The absorbance was measured at 450 nm in a plate reader.

### 4.5 Western blotting

Protein was extracted from frozen liver samples or cultured hepatocytes in cell lysis buffer. In total, 40 to 60 μg of protein was loaded onto a 10% SDS‐polyacrylamide gel, and separated proteins were transferred to PVDF membranes. The membranes were incubated with rabbit antibodies to β‐actin (1:1000; Santa Cruz), rabbit antibodies to Histone H3, E‐cadherin, ZO‐1, Fibronectin, Vimentin, (1:1000; CST), rabbit antibody to HMGB2 (1:1000; Abcam), overnight, and followed by HRP‐conjugated secondary antibody (1:10000; CST), respectively. The signal was detected using an enhanced chemiluminescence detection system (Pierce, Rockford, IL) as described by the manufacturer.

### 4.6 RNA‐pulldown analysis and RIP assay

The biotinylated LINC00341, antisense LINC00341, or mutated LINC00341 was mixed with proteins obtained from cancer cells for overnight at 4°C. The complex of biotinylated lncRNA and proteins was purified using streptavidin‐agarose for 4 hours at 4°C. The proteins are then eluted from the RNA‐protein complex and detected by Western blotting analysis. RIP assay was performed using RIP RNA‐binding protein immunoprecipitation kit (Merck Millipore) and following the manufacturer's protocol.

### 4.7 Statistical analysis

Data were analysed using GraphPad Prism 5. Differences between two comparisons were evaluated using Student's *t* test. Analysis of variance method was performed to analyse the differences in multiple comparisons. Log‐rank analysis was applied in survival comparisons. Statistical significance was established at *P* < .05.

## CONFLICT OF INTEREST

The authors declare no competing financial interests.

## AUTHOR CONTRIBUTIONS

S.L. wrote the manuscript and operated the experiments; S.C. and B.W. collected clinical samples and operated molecular experiments; X.L.Z. and Y.S. analysed the data; X.Z. designed the experiments and edited the manuscript.

## Data Availability

My article contains data are available
